# Metal Allergy and Systemic Contact Dermatitis: An Overview

**DOI:** 10.1155/2012/749561

**Published:** 2012-05-30

**Authors:** Yoko Yoshihisa, Tadamichi Shimizu

**Affiliations:** Department of Dermatology, Graduate School of Medicine and Pharmaceutical Sciences, University of Toyama, Toyama 930-0194, Japan

## Abstract

Contact dermatitis is produced by external skin exposure to an allergen, but sometimes a systemically administered allergen may reach the skin and remain concentrated there with the aid of the circulatory system, leading to the production of systemic contact dermatitis (SCD). Metals such as nickel, cobalt, chromium, and zinc are ubiquitous in our environment. Metal allergy may result in allergic contact dermatitis and also SCD. Systemic reactions, such as hand dermatitis or generalized eczematous reactions, can occur due to dietary nickel or cobalt ingestion. Zinc-containing dental fillings can induce oral lichen planus, palmoplantar pustulosis, and maculopapular rash. A diagnosis of sensitivity to metal is established by epicutaneous patch testing and oral metal challenge with metals such as nickel, cobalt, chromium, and zinc. *In vitro* tests, such as the lymphocyte stimulating test (LST), have some advantages over patch testing to diagnose allergic contact dermatitis. Additionally, the determination of the production of several cytokines by primary peripheral blood mononuclear cell cultures is a potentially promising *in vitro* method for the discrimination of metal allergies, including SCD, as compared with the LST.

## 1. Introduction

Contact dermatitis is usually produced by external exposure of the skin to an allergen; however, sometimes a systemically administered allergen may reach the skin through the circulatory system and thereby produce systemic contact dermatitis. Systemic contact dermatitis (SCD) is an inflammatory skin disease that is known to occur with exposure to drugs, foods, and dental metals. A variety of types of skin eruptions have been reported, including flares of previous patch test sites, symmetrical intertriginous and flexural exanthema, exfoliative erythroderma, and widespread dermatitis [1].

Metals such as nickel, cobalt, chromium, and zinc are ubiquitous in our environment. During the 20th century, industrialization and modern living resulted in increased cutaneous exposure to these metals and hence an increased incidence of metal allergies [2]. Metal allergies may result in allergic contact dermatitis. Metals that are electrophilic have the ability to ionize and react with proteins, thus forming complexes that can be recognized by dendritic cells, which allows for sensitization to occur [3]. Cases of contact dermatitis caused by cutaneous exposure to cosmetics products and jewelry that contain nickel have been reported in the literature. The thinness of the stratum corneum and intermittent exposure to sweat on the eyelids have been associated with increased nickel absorption through the skin from cosmetics, allowing lower nickel concentrations to elicit a reaction [4]. Cobalt is a strong skin sensitizer [5]. Over the years, occupational exposure to cobalt has been primarily observed in metal workers, bricklayers, and pottery workers. Contact dermatitis that results from direct contact to an allergen is the most common and easiest form of metal allergy to identify. However, the timely recognition of the type of systemic skin inflammation known as SCD and its varying presentations is critical as it can result in more chronic and severe symptoms.

## 2. Metals and SCD

### 2.1. Nickel and SCD

Nickel is a chemical element found ubiquitously in the environment and is used with a high frequency worldwide. This metal is manufactured into steel and a variety of alloys containing cobalt, palladium, iron, titanium, gold, and magnesium [6]. Sensitized individuals generally experience a predictable localized response following cutaneous exposure to nickel, including erythema, vesicle formation, scaling, and pruritus. According to recent studies, females have an about 4-fold higher relative risk of developing allergic contact dermatitis to nickel compared with males [6].

Systemic reactions, such as generalized eczematous reactions or dyshidrotic hand eczema, can occur due to dietary ingestion of nickel. In 1984, Andersen et al. coined the term “baboon syndrome” to describe the generalized dermatitis of the buttocks, anogenital area, flexures, and eyelids that is frequently observed in patients with SCD to nickel [7]. Nickel is present in most dietary items, and food is considered to be a major source of exposure to nickel for the general population. Certain foods are routinely found to be high in nickel content. Nickel present in the diet of a nickel-sensitive person can provoke SCD. For example, SCD can be elicited in nickel-sensitive individuals from the consumption of foods with a high nickel content, such as cocoa [8]. In such patients, adherence to a low-nickel diet and avoidance of local exposure to metal objects result in the disappearance of skin symptoms. Silvestri and Barmettler reported the case of a nickel-sensitive patient with a 1.5 year history of treatment-resistant pruritus ani [9]. The patient disclosed a habit of daily peanut butter consumption. His symptoms resolved with a restriction of dietary nickel [9]. A study of systemic nickel allergy found a dose-response relationship between nickel ingestion and the occurrence of dermatitis flare-ups [10]. Of note, for most nickel-allergic patients, a single dose of 4 mg of nickel will result in widespread dermatitis [10]. It is recommend that individuals with food-related flare-ups of nickel dermatitis consume a low-nickel diet [11].

Nickel is considered to be the most frequent contact allergen for patients with AD [12]. A recently published study of a German population showed a positive association between filaggrin mutations, which have been shown to be strongly associated with AD, and contact sensitization to nickel [13]. Another study also reported a positive association between nickel sensitization and AD, in a subanalysis of nonpierced women [14].

It is necessary to be aware of the systemic reactions that occur with SCD, which can be chronic and can produce severe symptoms that may often be mistaken for AD [15]. Initially, Shanon reported that patients with SCD occasionally experience a skin manifestation similar to AD called “pseudoatopic dermatitis” [16]. Hsu et al. recently reported four cases of children with variable presentations of SCD to nickel [15]. For each of these patients, the presence of clinically relevant exposure to nickel was confirmed with dimethylglyoxime testing. One of these patients, 16 years old, had a nine-year history of pruritic dermatitis that began on her infraumbilical area and arms. During the past year, the dermatitis had spread to the remainder of her body, including her face, and the patient was thus believed to have AD [15].

### 2.2. Cobalt and SCD

Although nickel sensitivity is more common than cobalt sensitivity, the two are frequently linked. Rystedt and Fischer reported that a quarter of nickel-sensitive patients developed a cobalt allergy and patients with simultaneous nickel and cobalt allergies have more severe dyshidrotic eczema [17]. It was proposed that a low-cobalt-diet reduced the dyshidrotic eczema flares in cobalt allergic patients [18]. Therefore, the ingestion of increased amounts of cobalt through food should be added to the list of triggering factors for SCD.

Furthermore, cobalt is contained in a variety of materials. Hard metal is manufactured by means of a powder metallurgical process in which about 90% tungsten carbide, small amounts of other metal carbides, and polyethylene glycol are mixed with about 10% metallic cobalt, which is used as a binding agent. Hard metal manufacturing involves pressing, forming, sintering, grinding, and etching or color marking. Cobalt exposure via inhalation may lead to cobalt-related asthma. Hard metal workers may develop cough, wheezing, and dyspnea that often improve during weekends and holidays [18]. The occurrence of localized contact dermatitis due to occupational exposure to cobalt in the hard metal industry has also been reported [19, 20].

However, contact with a hard metal powder in the workplace is a rare cause of SCD. In particular, there has been only one report of occupational cobalt-induced SCD [21]. The case was a 19-year-old male who had worked as a grinder for 1 month in a hard metal factory. The hard metals used in the factory contained cobalt. The patient developed erythema on his hands 2 weeks after starting the work, which thereafter progressed to a generalized eczematous eruption with itching [21]. Patch testing showed positive reactions for 1% cobalt chloride. After changing his workplace, his skin rush disappeared. In this case, the recurrent eczematous lesions of the hands were associated with a flare of systemic dermatitis and were highly suggestive of SCD triggered by cobalt inhalation. Dermatologists should, therefore, remind such patients to pay increased attention to avoid all kinds of cobalt exposure in their daily life and work.

### 2.3. Chromium and SCD

The element chromium was discovered by Vaquelin in 1798. It is ubiquitous in the environment and is widely used in the plating, leather tanning, pigmentation, dye production, metallurgy, and chemical industries and is found in cement as a byproduct of the cement manufacturing process itself [22, 23]. When exposed to skin, chromium salts can induce cutaneous irritation, which may progress to SCD in cases of chromium hypersensitivity [24]. Chromate-induced SCD is primarily exacerbated by skin contact with hexavalent and trivalent chromium compounds [25]; however, the ingestion of the allergen in the dichromate form has also been reported to cause exacerbations [26–29]. The oral ingestion of trivalent chromium, that is, chromium picolinate, for nutritional supplementation has been reported to cause SCD [30]. Recently, SCD resulting from the ingestion of chromium chloride in a multivitamin/multimineral tablet has been reported [31].

Metal allergy has also been associated with device failures following the insertion of intracoronary stents, hip and knee prostheses, and other implants. Gao et al. reported a case of SCD most likely caused by exposure to chromium after a total knee arthroplasty, although this complication is very rare [32]. The majority of total joint prostheses are now made of cobalt-chromium alloys with a nickel content of less than 1% [33]. The occurrence of SCD is particularly uncommon following total knee arthroplasty because there is a polyethylene insert between the femoral and tibial components and no metal-on-metal contact exists.

### 2.4. Zinc and SCD

**Figure 1:**
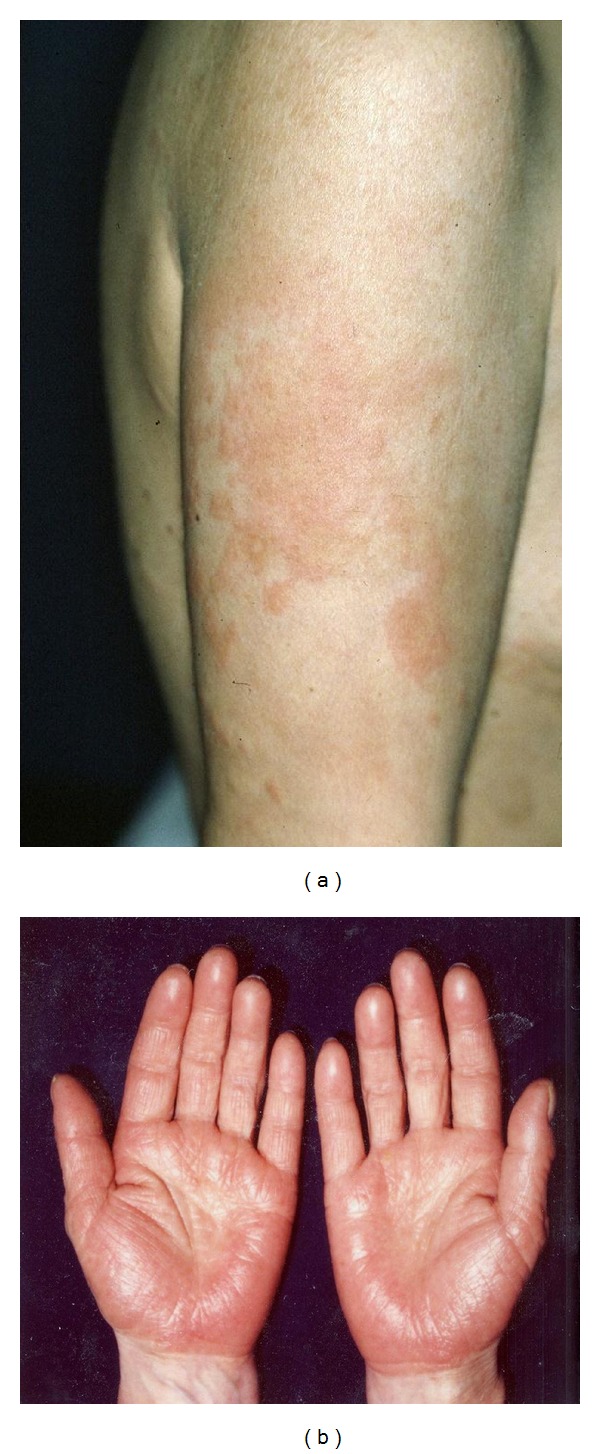
(a) A 49-year-old Japanese female with a diffuse edematous erythema with papules over her entire body. (b) The oral challenge test with zinc sulfate caused exacerbation of the preexisting eruptions on her palms, including itching edematous erythema.

Zinc is an essential trace element involved in many physiological functions, including catalytic and structural roles in metalloenzymes, as well as regulatory roles in diverse cellular processes, such as synaptic signaling and gene expression. Zinc is widely used in dental restoration. The previously reported dental metal eruptions caused by zinc have included oral lichen planus [34], palmoplantar pustulosis [35], and a maculopapular rash [36]. It has also been reported to cause severe symptoms in cases of SCD. One case was a 49-year-old Japanese female who developed facial edema, blepharedema, and pruritic edematous erythema with papules over her entire body. Based on the results of a metal patch test, lymphocyte stimulating test (LST), and zinc challenge test, a diagnosis of zinc-allergic SCD was made (Figure 1) [37]. This case had four teeth that had been treated with metal fillings, which likely contained zinc. All of the patient's dental fillings were completely removed, and her diet was changed to a zinc-restricted diet. Two weeks later, the majority of the skin lesions, which had lasted for four months, subsided rapidly [37].

 Saito et al. reported another severe case of SCD that developed because of the zinc contained in dental fillings, in which generalized flare-up reactions occurred from a zinc patch test [38]. In this case, one may suspect the amount of zinc that can be absorbed through the skin or oral mucosa compared with that obtained through dietary zinc intake to be small.

## 3. Diagnosis of Metal Sensitivity

Epicutaneous path testing has been used to diagnose metal sensitivity. It is the primary tool to diagnose allergens that cause allergic contact dermatitis. The main advantages of patch tests are that they can be completed without hospital surveillance since they rarely induce adverse reactions. Therefore, a patch test evaluation is the gold standard for detecting metal hypersensitivity. However, the accuracy of this method is strongly dependent on the experience of the observer, and distinguishing doubtful-positive from positive patch test reactions for different reagents remains difficult. Sometimes false-positive and negative reactions are observed in conditions of existing dermatitis. Some patch test substances, such as cobalt, nickel, copper, and chromium, sometimes cause false-positives and pustule formation [39, 40].

Oral metal challenges with nickel, cobalt, chrome, and zinc are sometimes performed and are diagnostic for metal allergies, especially SCD. However, flare-up reactions sometimes appear at previous sites of eczema, including hand eczema, and at patch test sites after an oral challenge [41].

*In vitro* tests, such as the LST, have some advantages over patch testing to diagnose allergic contact dermatitis. First, the LST does not cause flare-ups or exacerbation of symptoms in patients, is objective, and can be used in clinical situations where patch testing is not recommended [42]. However, the LST has not yet been sufficiently optimized or validated to be used as the sole routine diagnostic method for confirming a suspicion of a contact allergy. With regard to the diagnosis of nickel allergy, the task is made quite difficult because of the frequent overlap in test results between nickel-allergic and nonallergic subjects, which may be partly due to a nonspecific, mitogenic effect exerted by nickel [43].

It is useful to assess metal-induced cytokine profiles using the *in vitro *stimulation of primary peripheral blood mononuclear cells (PBMCs) with metal salts alone. Stimulation with nickel, cobalt, and chromium leads to a specific pattern of cytokine secretion in PBMC cultures obtained from metal-allergic patients, which involves both Th1- and Th2-type cytokines [44–47]. Based on a blood analysis of 14 patients with SCD to nickel, IFN-*γ* and IL-5 seem to play an important role in the pathogenesis of SCD [48]. Studies of the relationship between zinc and cytokines showed that zinc increased monokine secretion more efficiently than other related divalent cations, including cobalt, nickel, and mercury [49]. Furthermore, zinc stimulation of the PBMCs obtained from SCD patients showed higher macrophage migration inhibitory factor (MIF) and TNF-*α* secretion compared to that found in healthy subjects [37]. MIF increases TNF-*α* production and is thought to play an important role in contact hypersensitivity responses [50]. MIF is secreted from both Th1- and Th2-type cells [51]. This suggests that the presence of zinc in the peripheral blood of zinc-allergic patients induces PBMCs to produce increased levels of MIF, which could lead to SCD.

In conclusion, the determination of Th1- and Th2-type cytokine production in PBMC cultures is a potentially promising *in vitro* method for diagnosing metal allergies, including SCD. Therefore, the analysis of PBMC cultures may be helpful in confirming the diagnosis of SCD caused by metal allergy in patients with positive patch testing.
